# Characterizing unextendible product bases in qutrit-ququad system

**DOI:** 10.1038/srep11963

**Published:** 2015-07-14

**Authors:** Ying-Hui Yang, Fei Gao, Guang-Bao Xu, Hui-Juan Zuo, Zhi-Chao Zhang, Qiao-Yan Wen

**Affiliations:** 1 State Key Laboratory of Networking and Switching Technology, Beijing University of Posts and Telecommunications, Beijing, 100876, China; 2 School of Mathematics and Information Science, Henan Polytechnic University, Jiaozuo, 454000, China; 3 Mathematics and Information Science College, Hebei Normal University, Shijiazhuang, 050024, China

## Abstract

Unextendible product bases (UPBs) play an important role in quantum information theory. However, very little is known about UPBs in Hilbert space of local dimension more than three. In this paper, we study the UPBs in qutrit-ququad system and find that there only exist six, seven and eight-state UPBs. We completely characterize the six-state and seven-state UPBs. For eight-state UPBs, seven classes of UPBs are found. As auxiliary results, we study the distinguishability of qutrit-ququad UPBs by separable measurements, and find that there exists a UPB that cannot be distinguished.

The notion of unextendible product bases (UPBs) plays an important role in the theory of quantum information. It was first introduced by Bennett *et al.*^1^ to construct bound entangled states. A UPB is an orthogonal product basis whose complementary subspace contains no product states. A complete orthogonal product basis can also act as a UPB, but it is trivial. It is well known that the members of any nontrivial UPB cannot be perfectly discriminated by local operations and classical communication (LOCC)^1^, which exhibits the phenomenon “quantum nonlocality without entanglement”^2^. In addition, Duan *et al.* constructed locally indistinguishable subspaces with dimension four using UPB in three-qubit system^3^.

Although many important results have been reported (see Refs 1, 2, 3, 4, 5, 6, 7, 8, 9, 10, 11, 12, 13, 14, 15, 16, 17, 18, 19 for an incomplete list) during the past two decades, UPBs have only been completely characterized in limited cases (there are 0, 1, 1, 1446 nontrivial UPBs in 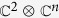, 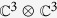, 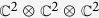 and 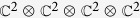, respectively^1^,^4^,^5^,^6^). The structure of UPBs in more complicated system is still not clear.

In this paper, we focus on nontrivial UPBs in 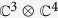, and try to present all of them. We find that there only exist six, seven and eight-state UPBs. We completely characterize the six-state and seven-state UPBs. For eight-state UPBs, seven classes of UPBs are found. Finally, we study the distinguishability of UPBs by separable measurements as auxiliary results.

## Results

### The orthogonality graph of an orthogonal product basis

An orthogonality graph is a very useful tool to investigate UPBs.

Let 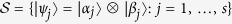 be an orthogonal product basis in bipartite system 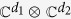. 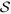 is represented by a graph *G* = (*V*, *E*_1_ 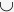 *E*_2_), where *V* is the set of vertices and *E*_*i*_ is the set of edges with color *i*. The states 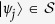 are represented as the vertices v_j_ ∈ *V*. There exists an edge *e* of color *i* between vertices *v*_*k*_ and *v*_*l*_, *i.e.*, *e* ∈ *E*_*i*_, if and only if states 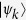 and 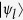 are orthogonal on *i*-th party. In the whole paper, every edge *e* ∈ *E*_1_ is represented by red solid line, and every edge *e* ∈ *E*_2_ is represented by black dotted line in orthogonality graphs.

The degree of *v*, denoted by *deg*(*v*), is the number of edges incident with *v*. In the orthogonality graph, the red degree of *v*, denoted by r-*deg*(*v*), is the number of red edges incident with *v*. Similarly, we can also define the black degree of *v*, denoted by b-*deg*(*v*).

**Figure 1:**
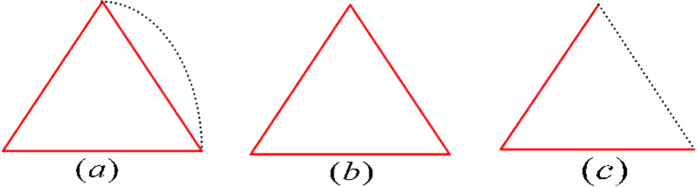
Orthogonality graph containing multiple edges for (**a**). Orthogonality graph (**a**) can be broken down into simple complete graphs (**b**) and (**c**). Red solid lines represent the orthogonality between different states on Alice’s side, and black dotted lines represent the orthogonality between different states on Bob’s side.

Note that there may be two edges of different colors between two different vertices when the two states are orthogonal for both parties. That is, the graph maybe contains multiple edges. If the orthogonality graph does not contain multiple edges, the orthogonality graph is a simple complete graph since all the states in 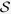 are mutually orthogonal. The graph containing multiple edges is more complex. So we break down the orthogonality graph containing multiple edges into several simple complete graphs. For example, Fig. 1. Suppose that a graph *G* with *n* multiple edges is an orthogonality graph of set 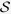, then *G* can be broken down into 2^*n*^ simple complete graphs 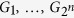. Obviously, if we can determine the extendibility of one orthogonality graph *G*_*i*_, then 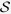 is extendible. In other words, if 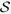 is a UPB, according to every orthogonality graph *G*_*i*_ we can always obtain that the set 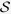 is a UPB. So the graphs with multiple edges can always be converted into the orthogonality graphs without multiple edges. And in orthogonality graphs without multiple edges, obviously, we have r-*deg*(*v*) + b-*deg*(*v*) = *deg*(*v*).

Now we recall a useful lemma^1^, which can determine whether an orthogonal product basis is a UPB or not.

LEMMA 1. *Let* 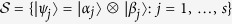 *be an orthogonal product basis in bipartite system* 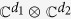. *Let P be a partition of* 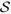 *into two disjoint subsets*: 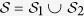. *Let* 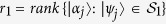, 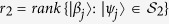. *Then* 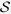 *is extendible if and only if there exists a partition P such that for all i* = 1,2, *the local rank r*_*i*_ *less than the local dimension d*_*i*_*, i.e., r*_*i*_ < *d*_*i*_.

If different UPBs have the same orthogonality graph up to relabeling the vertices (*i.e.*, these orthogonality graphs are isomorphic), we say these UPBs are equivalent. It is worth noting that a set of orthogonal product states, entirely come from the orthogonality graph of UPB, may not be a UPB, which may lead to unequivalence between UPB and this set^4^. However, different UPBs from the same orthogonality graph are equivalent. Note that UPBs which we refer to in the whole paper are all nontrivial.

To be explicit, when we refer to the ‘size’ of a UPB, we mean the number of states in the set of UPB. The minimum size of UPBs has been studied in Refs 7, 8, 9. The minimum size of UPBs is *d*_1_ + *d*_2_ – 1 in 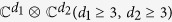 if and only if at least one of the two numbers *d*_1_, *d*_2_ is odd^7^. In addition, UPBs can always be used to construct bound entanglement. While there exists no bound entangled state with rank less than or equal to three^10^,^11^. According to the two results we have the following lemma:

LEMMA 2. *In* 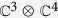 *system, the minimum size of UPBs is six, and the maximum size is eight*.

We are now ready to present all of the nontrivial UPBs in 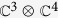. In the sense of the same orthogonality graph, we show the orthogonality graphs of UPBs and their mathematical structures.

### Six-state UPBs

**Figure 2:**
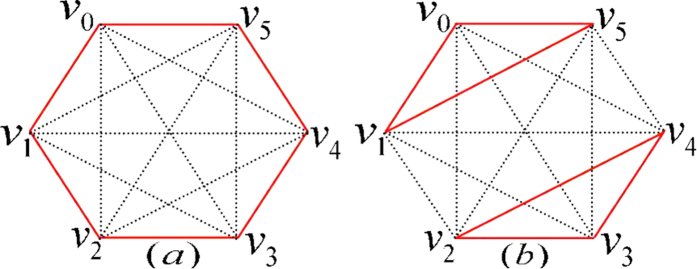
Six-state orthogonality graph with r-*deg*(*v*_i_) = 2 and b-*deg*(*v*_i_) = 3, 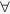
***i***. Red solid lines represent the orthogonality between different states on Alice’s side, and black dotted lines represent the orthogonality between different states on Bob’s side.

THEOREM 1. *All the six-state UPBs must have the same orthogonality graph as* *Fig. 2(a)*, *conversely, the sets of orthogonal product states corresponding to orthogonality graph* *Fig. 2(a)* *are all UPBs. Furthermore, all the six-state UPBs can be characterized by the states* 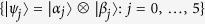 *(up to some local unitary):*


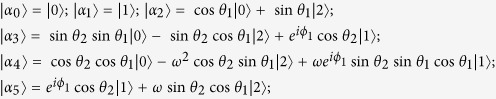



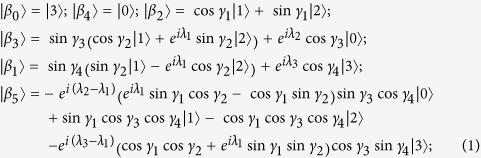


*where* 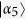 *and* 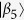 *are not normalized. And* 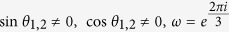, sin γ_3,4_ ≠ 0, cos γ_3,4_ ≠ 0, 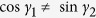 (or *λ*_1_ ≠ *π*), *and* 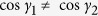 (*or λ*_1_ ≠ 0).

*Proof*. We can prove the result from two aspects. On the one hand, any UPB with six states must have the same orthogonality graph Fig. 2(a). In other words, the sets 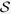 corresponding to orthogonality graph with six vertices are always extendible except for Fig. 2(a). If there exists a vertex which is connected to five (*i.e.*, 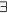
*i*, r-*deg*(*v*_*i*_) = 5 or b-*deg*(*v*_*i*_) = 5) or four (*i.e.*, 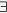
*i*, r-*deg*(*v*_*i*_) = 4 or b-*deg*(*v*_*i*_) = 4) other vertices with edges of same color in orthogonality graph of 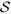, it is easy to verify that the set 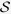 is extendible. It is because the partition in lemma 1 is the five or four states as the set 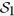 (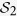) and the other states as 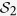 (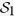). If there exists a vertex which is connected to three other vertices with red edges, *i.e.*, 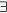
*i*, r-*deg*(*v*_*i*_) = 3, without loss of generality, suppose that vertex *v*_0_ is connected to *v*_1_,*v*_2_ and *v*_3_ with red edges. It means that the state 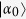 is orthogonal to 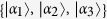 on Alice’s side. Then *rank*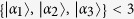 (because local dimension is three on Alice’s side). While *rank*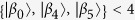 on Bob’s side. Applying lemma 1, the set 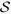 is extendible. Now all vertices have to be connected to exactly two other vertices with red edges and three other vertices with black edges. Namely, 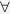
*i*, r-*deg*(*v*_*i*_) = 2 and b-*deg*(*v*_*i*_) = 3.

It is straightforward to see that there are only two possible orthogonality graphs Fig. 2(a,b). Considering Fig. 2(b), on Bob’s side every vector in the set 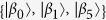 is orthogonal to every vector in the set 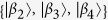, thus he can distinguish the two sets by local projection measurements. Then Alice can discriminate the remaining three states on Alice’s side based on Bob’s measurements result. It means that the set 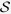 corresponding to Fig. 2(b) can always be distinguished by LOCC. So it is extendible since UPBs cannot be distinguished by LOCC. In a word, any six-state UPB must have the same orthogonality graph Fig. 2(a).

On the other hand, the sets of orthogonal product states entirely from orthogonality graph Fig. 2(a) are UPBs. First of all, we show that on Alice’s side the rank of any three different states must be three. If there exist three states whose rank is one, the three states must be 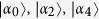 or 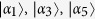 (since two adjacent vertices are orthogonal each other). Without loss of generality, assume that the three states are 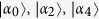, then 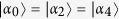 (up to the overall phase). Since 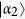 is orthogonal to 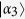, we have 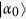 is also orthogonal to 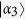. That is, *v*_0_ is connected to *v*_3_ with red edges. It contradicts with Fig. 2(a).

If there exist three different states of which rank is two, then one of the three states must be expressed as a linear combination of the two remaining states. All the cases can be proved using similar method, without loss of generality, assuming that *rank*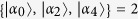, and 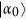 can be expressed as a linear combination of 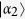 and 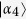. While 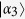 is orthogonal to 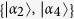, then 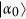 is also orthogonal to 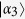. It contradicts with Fig. 2(a). In one word, on Alice’s side the rank of any three different states must be three.

Now we show that on Bob’s side the rank of any four different states must be four. The similar discussion on Alice’s side can be applied here. It can be easily analyzed that the rank of any two states is two, and the rank of any three states is three. Otherwise it contradicts with the Fig. 2(a). Suppose that there exist four states of which the rank is three. (i) If the four states are adjacent, without loss of generality, assume *rank*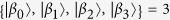, then 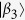 can be expressed as a linear combination of 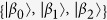. Since 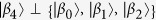, we have 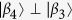. It contradicts with Fig. 2(a). (ii) If three of four states are adjacent, without loss of generality, assume *rank*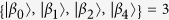. It is impossible that the rank is three because *rank*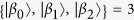 and 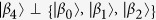. (iii) If two of four states are adjacent, assume *rank*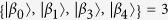. Since *rank*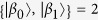, 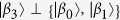, and 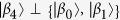, we have 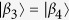. It contradicts that the rank of any two states is two. So on Bob’s side the rank of any four different states must be four.

Overall, in any set of orthogonal product states entirely from orthogonality graph Fig. 2(a), on Alice’s side the rank of any three different states must be three and on Bob’s side the rank of any four different states must be four. Therefore, the sets from Fig. 2(a) are UPBs by lemma 1.

Furthermore, according to the orthogonality graph Fig. 2(a), we can easily construct all the six-state UPBs Eq. (1). The conditions under Eq. (1) ensure that there does not exist another orthogonal relationship except for orthogonality in orthogonality graph.
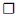


It should be noted that there does not exist a six-state UPB whose orthogonality graph contains multiple edges. Because according to the proof every vertex in orthogonality graph have to be connected to exactly two vertices with red edges and three other vertices with black edges. Namely, 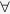
*i*, r-*deg*(*v*_*i*_) = 2 and b-*deg*(*v*_*i*_) = 3. The orthogonality graph of six-state UPBs is unique, which is Fig. 2(a).

### Seven-state UPB

GenTiles2 is a class of UPBs^4^ in 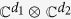 (*d*_1_ ≥ 3, *d*_2_ ≥ 4), and the size of the UPBs is *d*_1_*d*_2_ − 2*d*_1_ + 1. When *d*_1_ = 3, *d*_2_ = 4, it is a seven-state UPB in 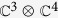. The seven states are as follows:


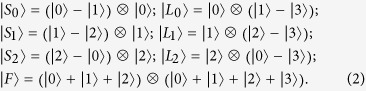


**Figure 3:**
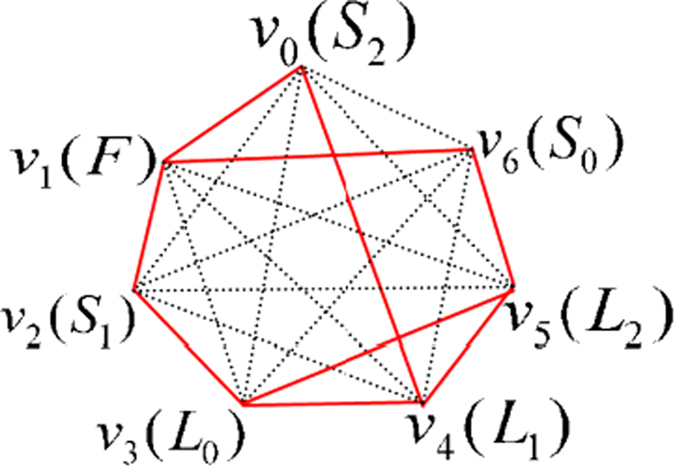
Seven-state orthogonality graph of UPBs.

Its orthogonality graph is Fig. 3.

Next we will prove that any UPB with seven states must have the only orthogonality graph, *i.e.*, Fig. 3. In other words, the set 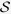 corresponding to orthogonality graph with seven vertices are always extendible except for Fig. 3. We first prove a simple lemma.

LEMMA 3. *In orthogonality graph with seven states, it is impossible that the number of vertices with r-deg*(*v*_*i*_) = 2, *b-deg*(*v*_*i*_) = 4 *is even and other vertices with r-deg*(*v*_*i*_) = 3*, b-deg*(*v*_*i*_) = 3.

*Proof.* Assuming that there are *x* vertices with r-*deg*(*v*_*i*_) = 2, b-*deg*(*v*_*i*_) = 4, *x* is even. Then the red degree of all the vertices is 2*x* + 3(7 − *x*). Obviously, 2*x* + 3(7 − *x*) is odd. It contradicts that the red degree must be twice the number of red edges.
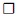


THEOREM 2. *All the seven-state UPBs must have the same orthogonality graph as* *Fig. 3* *in* 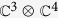.

*Proof*. We need only prove that the sets 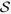 corresponding to orthogonality graph with seven vertices are always extendible except for Fig. 3. If there exists a vertex which is connected to six (*i.e.*, 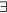
*i*, r-*deg*(*v*_*i*_) = 6 or b-*deg*(*v*_*i*_) = 6) or five (*i.e.*, 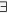
*i*, r-*deg*(*v*_*i*_) = 5 or b-*deg*(*v*_*i*_) = 5) other vertices with edges of same color in orthogonality graph of 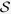, obviously, the set 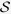 is extendible by lemma 1. If there exists a vertex which is connected to four other vertices with red edges, *i.e.*, 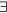
*i*, r-*deg*(*v*_*i*_) = 4, it is easy to prove that the set 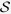 is extendible. Now we need only consider that 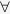
*i*, r-*deg*(*v*_*i*_) = 2, b-*deg*(*v*_*i*_) = 4 or r-*deg*(*v*_*i*_) = 3, b-*deg*(*v*_*i*_) = 3. According to lemma 3, the number of vertices with r-*deg*(*v*_*i*_) = 2, b-*deg*(*v*_*i*_) = 4 is 1, 3, 5 or 7. In the light of lemma 2, 3, 5 and 7 in supplementary information, we know that the set 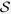 corresponding to orthogonality graph with seven vertices are always extendible except for Fig. 3. That is, all the seven-state UPBs must have only orthogonality graph Fig. 3.
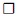


THEOREM 3. *The sets of orthogonal product states corresponding to orthogonality graph Fig. 3 are all UPBs*.

*Proof*. For simplicity, we first construct orthogonal product states 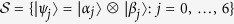 from orthogonality graph Fig. 3. Since 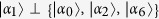 in Fig. 3, we know 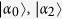 and 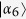 are linearly dependent. Then 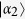 can be linearly expressed by 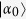 and 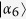, otherwise it contradicts with Fig. 3. We now start with the red triangle on Alice’s side to construct them (up to some local unitary):


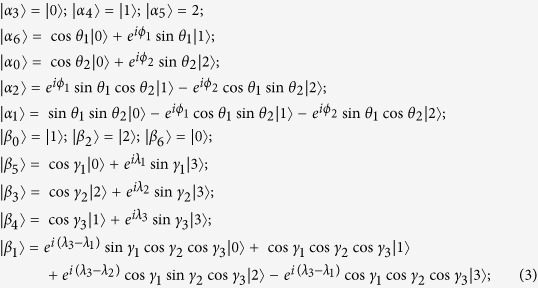


where 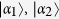 and 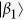 are not normalized. And on Alice’s side 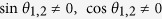, on Bob’s side 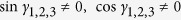. The range of these parameters ensures that the states in Eq. (3) are not orthogonal except for orthogonality in orthogonality graph.

We can easily verify the following features of these states in Eq. (3): (i) on Alice’s side the rank of any four states is three; (ii) on Bob’s side the rank of any five states is four; In order to find a partition 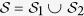 satisfying lemma 1, according to the above features we have 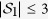 and 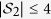. So 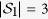 and 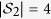. On Bob’s side, it is easy to verify that there are only three sets 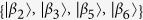, 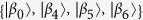 and 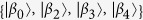 in Eq. (3) which are linearly dependent and can be as 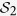. The three sets happen to be the neighbors of three vertices with b-*deg*(*v*_*i*_) = 4 respectively. However, on Alice’s side the three sets as 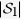, *i.e.*, 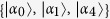, 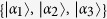 and 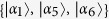 are all independent. Therefore, there does not exist the partition satisfying lemma 1. That is, the set of orthogonal product states from orthogonality graph Fig. 3 are UPBs.
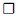


Employing theorem 2, 3, we can obtain the following theorem to completely characterize the seven-state UPBs.

THEOREM 4. *All the seven-state UPBs must have the same orthogonality graph as Fig. 3, conversely, the sets of orthogonal product states corresponding to orthogonality graph Fig. 3 are all UPBs. Furthermore, all the seven-state UPBs* 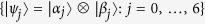 *can be characterized by Eq. (3)(up to some local unitary)*.

It should be noted that there is also no seven-state UPB whose orthogonality graph contains multiple edges. Suppose that there exists such a UPB 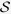, then we can break down the orthogonality graph containing multiple edges into some simple complete graphs which are the orthogonality graph of UPBs. However, Fig. 3 is the only orthogonality graph without multiple edges of UPB, and others are extendible. It is straightforward to verify that 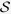 is extendible. It is a contradiction.

### Eight-state UPBs

A strongly uncompletable product basis (SUCPB)^4^ is a product basis spanning a subspace 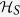 in a locally extended Hilbert space 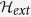 such that for all 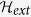 the subspace 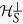 (
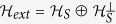
) contains fewer mutually orthogonal product states than its dimension. It means that a SUCPB can always be extendible to be a UPB.

**Figure 4:**
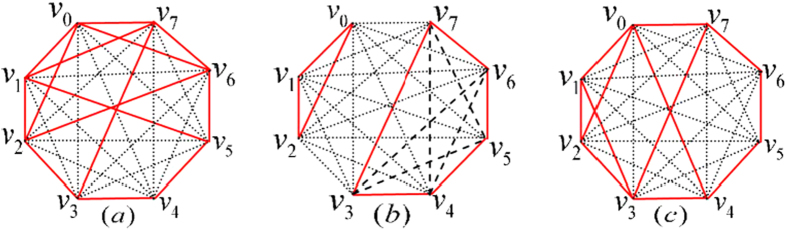
Eight-state orthogonality graph of UPBs without multiple edges.

**Pyr34**^**+**^ is a SUCPB^4^, which was used to construct bound entangled state in 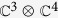. Then it can be extendible to be a UPB in 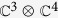, and the orthogonality graph of the UPB is Fig. 4(a). Another famous UPB in 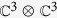 is the **Pyramid** UPB^1^,^4^ which can be as a SUCPB in 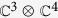 (since 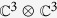 can be as a subspace in 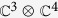). So it can be also extendible to be a UPB in 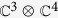. And the orthogonality graph of the UPB is Fig. 4(b).

THEOREM 5. *The sets of orthogonal product states entirely from orthogonality graph* Fig. 4(a) *are all UPBs*.

*Proof.* We can also construct the orthogonal product states 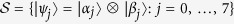 corresponding to Fig. 4(a). First we observe the characteristics of the graph Fig. 4(a). On Alice’s side considering the red squares *v*_0_*v*_1_*v*_7_*v*_6_ and *v*_1_*v*_2_*v*_0_*v*_7_, we can easily find that 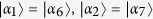, respectively. On Bob’s side since both 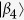 and 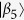 are orthogonal to 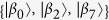, the three states 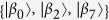 are linearly dependent and 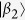 can be linearly expressed by 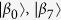, otherwise it contradicts with Fig. 4(a). We now construct the orthogonal product states corresponding to Fig. 4(a), and start with the red triangle *v*_0_*v*_1_*v*_2_ on Alice’s side and the black triangle *v*_1_*v*_4_*v*_6_ on Bob’s side.


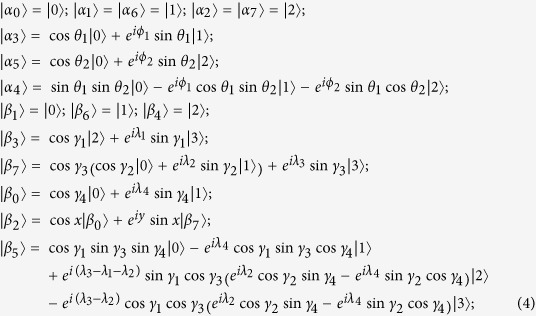


where *x*, *y* in 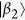 are solution of equation 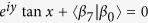, obviously 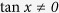, and 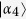, 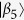 are not normalized. And on Alice’s side 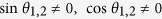, on Bob’s side 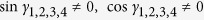 and 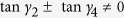.

From the Eq. (4), we can easily find that sets of orthogotanal product states from Fig. 4(a) has following features: on Alice’s side the rank of any five states are three; on Bob’s side the rank of any five states are four. So in order to find the partition satisfying lemma 1, we have 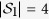 and 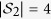. From the Eq. (4), we can also find that (i) on Alice’s side the rank of any four states are three except for the three sets 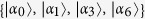, 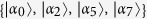, and 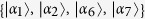. And the rank of the three sets are all two; (ii) However, on Bob’s side the rank of the three sets 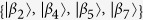, 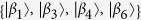, and 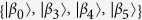 are all four, which are the sets corresponding to the three sets on Alice’ side. It means that there does not exist the partition satisfying lemma 1. So the sets corresponding to Fig. 4(a) are UPBs.
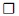


THEOREM 6. *The sets of orthogonal product states entirely from orthogonality graph* Fig. 4(b) *are all UPBs*.

*Proof*. From orthogonality graph Fig. 4(b), it is easy to see that 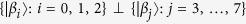. It means that *rank* 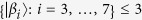. The states 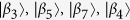 and 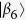 are orthogonal in turn. However, it is impossible that there exist odd number of states which are orthogonal in turn in two dimension space. Then the rank is not two. So 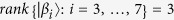. The states 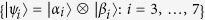 can degenerate in the system 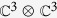. From the orthogonality graph of five states we know that they form a UPB in 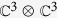 and can be as a SUCPB in 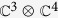. Thus they can be extendible to be a UPB in 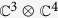. While the maximum size of UPB^4^ is eight in 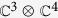, 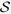 is a UPB, which comes from the extension of UPB in 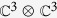.
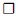


**Figure 5:**
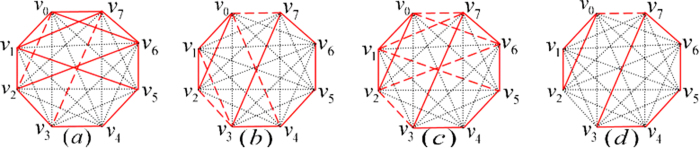
Eight-state orthogonality graph of UPBs with multiple edges. Long dashed red lines are multiple edges.

Now we can construct the set of this class of UPBs base on the UPB^4^ in 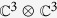.


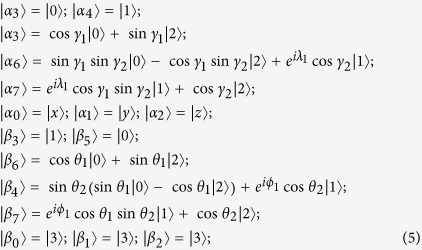


where 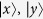 and 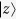 are any three mutually orthogonal states in three dimension space, but they cannot be orthogonal to 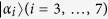. And 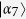, 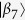 are not normalized. And 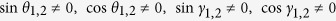. Note that in term of the different choices of 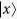,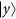 and 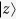, orthogonality graph maybe contains multiple edges. Fig. 4(b) is only an orthogonality graph without multiple edges, and orthogonality graph with multiple edges will be presented in Fig. 5.

THEOREM 7. *The sets of orthogonal product states entirely from orthogonality graph* Fig. 4(c) *are all UPBs*.

*Proof*. We first construct the orthogonal product states 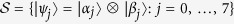 corresponding to Fig. 4(c). In the Fig. 4(c), considering the red square *v*_0_*v*_1_*v*_3_*v*_2_, we have 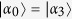. Next we prove 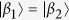. On Bob’s side 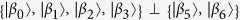. From the Fig. 4(c), it is easy to see 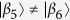. So 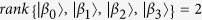. If 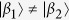, both 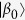 and 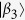 can be linearly expressed by 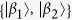. Since 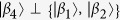, we have 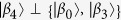. It is a contradiction. Thus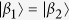. Now we begin to construct orthogonal product states.


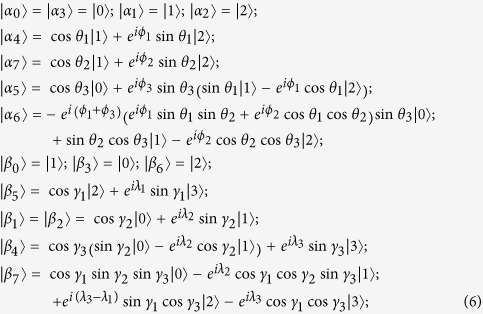


where 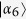 and 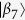 are not normalized. And 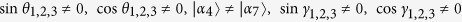.

It is easy to verify that the states on Alice’s side have the following features: (i) the rank of any five states is three; (ii) the rank of the only four states 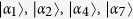 is two, and the rank of the other any four states is three; (iii) the rank of any three states in the set 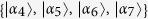 is three. The states on Bob’s side also have some features: (iv) the rank of the four states 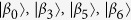 is four; (v) the rank of any five states which contain the four states 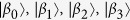 is three. And the rank of the other any five states is four; (vi) the rank of any six states is four.

Applying the above features (i)(vi), obviously, if the partitions for the set 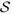 satisfy that 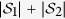 is 1 + 7, 2 + 6, 5 + 3, 6 + 2 or 7 + 1, the condition of lemma 1 cannot be satisfied. If the partitions satisfy that 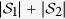 is 3 + 5, employing the features (iii)(v), the condition of lemma 1 cannot be satisfied, either. Finally, if the partitions satisfy that 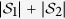 is 4 + 4, they do not satisfy lemma 1 applying the features (ii)(iv). Overall, there does not exist a partition satisfying lemma 1. Thus 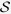 is a UPB.
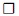


Through the analysis of a great number of the orthogonality graphs without multiple edges, we have the following conjecture.

CONJECTURE 1. *In the sense of the same orthogonality graph, eight-state UPBs whose orthogonality graphs do not contain multiple edges are only three, i.e.* Fig. 4(a–c).

Next we can use the orthogonality graphs without multiple edges to construct UPBs whose orthogonality graph contains multiple edges.

For the sake of simplicity, we denote the sets of orthogonal product states in Fig. 4(a–c) as 
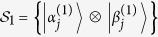
, 
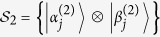
, 
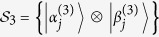
, respectively. According to the former discussion about orthogonality graph with multiple edges, the sets of orthogonal product states may be UPBs only if its orthogonality graph can be broken down into some of Fig. 4 in 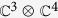. So we can construct the set satisfying the condition and then determine whether they are UPBs or not.

Employing Fig. 4(a,c), we can construct 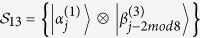, whose orthogonality graph is Fig. 5(a). Obviously, both Fig. 4(a,c) can be as their orthogonality graphs without multiple edges. It is easy to verify that 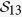 is a UPB. 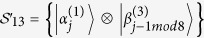 is also a UPB, whose orthogonality graph is isomorphic to Fig. 5(a). Similarly, we can also construct 
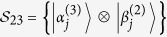
 and 
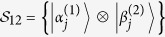
. Their orthogonality graphs are Fig. 5(b,c), respectively. It is straightforward to verify that both of them are UPBs. It should be noted that Fig. 4(a–c) can be all the orthogonality graphs without multiple edges of 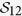. Let a set 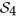 be a special form of 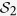, *i.e.*, in Eq. (5), 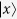 is only orthogonal to one of the vectors 
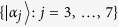
. Without loss of generality, assuming 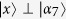, obviously, 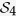 is a UPB whose orthogonality graph is Fig. 5(d). Now we have presented all the UPBs whose orthogonality graphs contain multiple edges applying Fig. 4. If the conjecture is right, there are only four different UPBs Fig. 5(a–d) with multiple edges in the sense of the same orthogonality graph.

### Distinguishability of UPBs by separable measurements

It is well known that all UPBs in 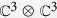 can be distinguished by separable measurements^4^. Recently, a UPB in 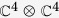 which cannot be distinguished by separable measurements was proved^20^. As auxiliary results, now we present the distinguishability of UPBs by separable measurements in 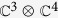.

THEOREM 8. *All the UPBs corresponding to* *Figs 2(a), 4(b) and 5(b–d)* *can be distinguished by separable measurements*.

THEOREM 9. *There is a UPB from* Fig. 4(a) *that cannot be distinguished by separable measurements*.

For the UPBs corresponding to Figs 3, 4(c) and 5(a), there are UPBs which can be distinguished by separable measurements. The proof of the two theorems and other discussions are given in supplementary information.

## Discussion

We have characterized the UPBs in 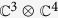. Specifically, both six-state UPBs and seven-state UPBs are only one in the sense of the same orthogonality graph, as Figs 2(a) and 3 depicted, respectively. We also present their mathematical structures. For eight-state UPBs, we find three classes of UPBs whose orthogonality graphs do not contain multiple edges, *i.e.*, Fig. 4(a–c). Meanwhile, we use them to construct the UPBs of orthogonality graphs with multiple edges, *i.e.*, Fig. 5(a–d). Finally, we present that the UPBs entirely from Figs 2(a), 4(b) and 5(b–d) can be distinguished by separable measurements, respectively. However there is a UPB from Fig. 4(a) which cannot be distinguished by separable measurements. We hope that these results will encourage researchers to develop the field further.

## Additional Information

**How to cite this article**: Yang, Y.-H. *et al.* Characterizing unextendible product bases in qutrit-ququad system. *Sci. Rep.* **5**, 11963; doi: 10.1038/srep11963 (2015).

## Supplementary Material

Supplementary Information
