# Supplementary material for: Characterizing unextendible product bases in qutrit-ququad system
Source: Sci Rep. 2015 Jul 14;5:11963. doi: 10.1038/srep11963 (PMC4500951; doi:10.1038/srep11963)
Supplement: Supplementary Information [file srep11963-s1.pdf]

Characterizing unextendible product bases in qutrit-ququad system
-Supplementary Information-

Ying-Hui Yang[1,2], Fei Gao[1,†], Guang-Bao Xu[1], Hui-Juan Zuo[1,3], Zhi-Chao Zhang[1], & Qiao-Yan Wen [1]

[1] *State Key Laboratory of Networking and Switching Technology, Beijing University of Posts and Telecommunications, Beijing, 100876, China*

[2] *School of Mathematics and Information Science, Henan Polytechnic University, Jiaozuo, 454000, China*

[3] *Mathematics and Information Science College, Hebei Normal University, Shijiazhuang, 050024, China*

THE PROOF OF LEMMA AND THEOREM

Proof of lemma. In order to prove theorem 2, we need prove the following lemma 2, 3, 5 and 7. In the process we use some basic notions in graph theory, which can easily find in Ref. [1]. And some simple results, *i.e.*, lemma 1, 4, 6, are proved. They can assist us to obtain the results which we need.

LEMMA 1. *In a simple graph with seven vertices, if there only exists one vertex with $deg(v_i) = 2$ and the other vertices with $deg(v_i) = 3$, the graph must contain a Hamiltonian cycle.*

Proof. We first show that the graph is connected. If it is disconnected, it has at least two connected components. Since $\forall i$, $deg(v_i) \geq 2$, every connected component has a cycle. Obviously, there are only two connected components and the connected component which has the minimum number of vertices have three vertices (since in a simple graph no more than two vertices cannot form a cycle). In the connected component with three vertices, the degree of every vertex is two. It is a contradiction.

Next we will construct a Hamiltonian cycle in the graph. Assume $deg(v_0) = 2$, we start with v_0 to construct a path P in the graph with maximum length (any two vertices in P are different), and say the vertices of P, in order, are $(v_0, v_1, \ldots, v_i)$. Obviously, $i \geq 5$, and neighbors of v_0 and v_5 are on P. (i) If $i = 5$, neighbors of v_6 can be $\{v_1, v_2, v_4\}$ or $\{v_1, v_3, v_4\}$ or $\{v_1, v_2, v_4\}$. Assuming that its neighbors are $\{v_1, v_3, v_4\}$, neighbors of v_5 are $\{v_0, v_2\}$. Thus there exists a Hamiltonian cycle $(v_0, v_1, v_2, v_3, v_6, v_4, v_5, v_0)$. Similar discussion to other cases. (ii) If $i = 6$, P is a Hamiltonian path. If v_0 is a neighbor of v_6, it forms a Hamiltonian cycle. Otherwise, the other neighbors of v_6 except for v_5 are two of four vertices $\{v_1, v_2, v_3, v_4\}$. Whatever the two vertices are, we can always find a Hamiltonian cycle. The method is very simple and similar, so we only consider one of the cases. Suppose that other neighbors of v_6 are $\{v_1, v_4\}$, then the neighbor of v_0 except for v_1 is v_2 or v_3. Therefore, the Hamiltonian cycles are $(v_0, v_2, v_3, v_4, v_5, v_6, v_1, v_0)$ or $(v_0, v_1, v_2, v_5, v_6, v_4, v_3, v_0)$ respectively. □

LEMMA 2. *The set $\mathcal{S}$ corresponding to orthogonality graph which only have one vertex with r-$deg(v_i) = 2$, b-$deg(v_i) = 4$ and the other six vertices with r-$deg(v_i) = 3$, b-$deg(v_i) = 3$ are always extendible.*

Proof. Since the graphs only have one vertex with r-$deg(v_i) = 2$, b-$deg(v_i) = 4$, there exists a red Hamiltonian cycle by lemma 1. Without loss of generality, assume that r-$deg(v_6) = 2$, b-$deg(v_6) = 4$, all the cases can be divided into four cases Fig. 1 in the sense of the same orthogonality graph.

Case 1 (Fig. 1(a)): Considering the red square $v_0v_1v_2v_3$, we have $|\alpha_0\rangle = |\alpha_2\rangle$ or $|\alpha_1\rangle = |\alpha_3\rangle$ (because on Alice's side its local dimension is three). In fact it is impossible that $|\alpha_0\rangle = |\alpha_2\rangle$. Suppose that $|\alpha_0\rangle = |\alpha_2\rangle$, then $|\alpha_2\rangle$ will orthogonal to $|\alpha_6\rangle$ since $|\alpha_0\rangle \perp |\alpha_6\rangle$. It contradicts with Fig. 1(a). So $|\alpha_1\rangle = |\alpha_3\rangle$. Similar discussion for the red square $v_2v_3v_4v_5$, we have $|\alpha_2\rangle = |\alpha_4\rangle$. Then $rank\{|\alpha_1\rangle, |\alpha_2\rangle, |\alpha_3, |\alpha_4\rangle\} \leq 2$. Since $rank\{|\beta_0\rangle, |\beta_5\rangle, |\beta_6\rangle\} \leq 3$, the set $\mathcal{S}$ is extendible.

Case 2 (Fig. 1(b)): Considering the red square $v_1v_3v_4v_2$, we have $|\alpha_1\rangle = |\alpha_4\rangle$. Then $|\alpha_1\rangle \perp |\alpha_5\rangle$ and $|\alpha_0\rangle \perp |\alpha_4\rangle$. It contradicts with Fig. 1(b). It means that Fig. 1(b) is not the orthogonality graph satisfying the conditions of lemma.

Case 3 (Fig. 1(c)): Considering the red square $v_1v_2v_3v_4$, we have $|\alpha_1\rangle = |\alpha_3\rangle$ or $|\alpha_2\rangle = |\alpha_4\rangle$. Whatever the subcase is, it always contradicts with Fig. 1(c).

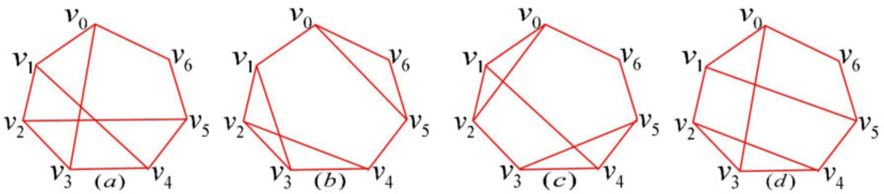

Figure 1: The graphs only have one vertex v_6 with r-$deg(v_6) = 2$, b-$deg(v_6) = 4$. In order to facilitate the observation, black dotted lines are not drawn.

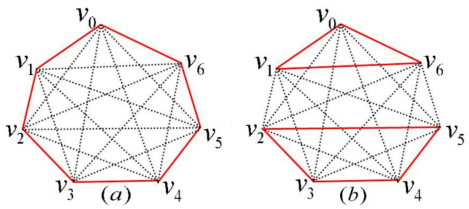

Figure 2: The graphs have seven vertex with r-$deg(v_i) = 2$, b-$deg(v_i) = 4$.

Case 4 (Fig. 1(d)): A similar discussion for the red square $v_1v_2v_4v_5$ to that in case 3 can be applied here.

According to the above discussion we know that Fig. 1(a) is the only orthogonality graph satisfying the conditions of lemma and the set $\mathcal{S}$ corresponding to Fig. 1(a) is extendible. □

LEMMA 3. *The set $\mathcal{S}$ corresponding to orthogonality graph which have seven vertices with r-$deg(v_i) = 2$, b-$deg(v_i) = 4$ are always extendible.*

Proof. Since in the graphs the degree of every vertex is r-$deg(v_i) = 2$, b-$deg(v_i) = 4$, the orthogonality graphs are only two cases Fig. 2(a)(b).

Case 1 (Fig. 2(a)): We first consider the linear relationship of three states $|\beta_0\rangle, |\beta_1\rangle, |\beta_2\rangle$ on Bob's side in Fig. 2(a). (i) They are linearly independent. Since $|\beta_4\rangle \perp \{|\beta_0\rangle, |\beta_1\rangle, |\beta_2\rangle\}$ and $|\beta_5\rangle \perp \{|\beta_0\rangle, |\beta_1\rangle, |\beta_2\rangle\}$, then $|\beta_4\rangle = |\beta_5\rangle$. Obviously $|\beta_5\rangle \perp |\beta_3\rangle$ in Fig. 2(a), so $|\beta_4\rangle \perp |\beta_3\rangle$. It contradicts with Fig. 2(a). (ii) They are linearly dependent. Then one of the three states can be expressed as a linear combination of the other states. If $|\beta_2\rangle$ can be expressed by a linear combination of $|\beta_0\rangle$ and $|\beta_1\rangle$, then $|\beta_3\rangle \perp |\beta_2\rangle$ since $|\beta_3\rangle \perp \{|\beta_0\rangle, |\beta_1\rangle\}$. It contradicts with Fig. 2(a). Otherwise, it means that $|\beta_2\rangle$ cannot be expressed by $|\beta_0\rangle$ and $|\beta_1\rangle$, *i.e.*, $|\beta_0\rangle = |\beta_1\rangle$. Since $|\beta_2\rangle \perp |\beta_0\rangle$, then $|\beta_2\rangle \perp |\beta_1\rangle$. It also contradicts with Fig. 2(a). So Fig. 2(a) is not the orthogonality graph satisfying the conditions of lemma.

Case 2 (Fig. 2(b)): The set $\mathcal{S}$ corresponding to Fig. 2(b) can always be distinguished by LOCC. Because Bob can first perform a von Neumann measurement to discriminate $\{|\psi_0\rangle\, |\psi_1\rangle\, |\psi_6\rangle\}$ and $\{|\psi_2\rangle\, |\psi_3\rangle\, |\psi_4\rangle, |\psi_5\rangle\}$. Since a set of orthogonal product states, which has four or fewer members, is distinguishable by LOCC in bipartite system [2], any one of two sets can always be distinguished by LOCC.

Therefore, the sets corresponding to orthogonality graph which have seven vertices with r-$deg(v_i) = 2$, b-$deg(v_i) = 4$ are all extendible. □

LEMMA 4. *In simple connected graph with seven vertices, if there exist five vertices with $deg(v_i) = 2$ and the other two vertices with $deg(v_i) = 3$, the graph must contain a Hamiltonian path.*

Proof. Let P be a path in the graph with maximum length (any two vertices on P are different), and say the vertices of P, in order, are $(v_0, v_1, \ldots, v_i)$. We will prove $i = 6$. Assuming $i < 6$, obviously, neighbors of v_0 and v_i are on P. (i) If v_i is a neighbor of v_0, the vertices on P form a circle. Since the graph is connected, the vertices which are not on P must be connected to some vertices on P. So there exists a path which is longer than P. It is a contradiction because P is a path with maximum length. (ii) If v_i is not a neighbor of v_0, there exist two vertices with degree 3 which are the neighbors of v_0 and v_i respectively. Since there are only two vertices with $deg(v_i) = 3$, the vertices which are not on P cannot be connected to the vertices on P. It contradicts that the graph is connected. Thus $i = 6$, *i.e.*, P is a Hamiltonian path. □

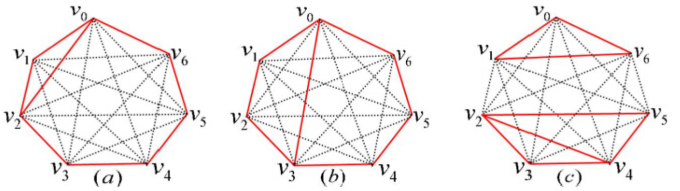

Figure 3: The graphs have five vertices with r-$deg(v_i) = 2$, b-$deg(v_i) = 4$. Graphs containing a Hamiltonian cycle for (a) and (b), disconnected graphs for (c).

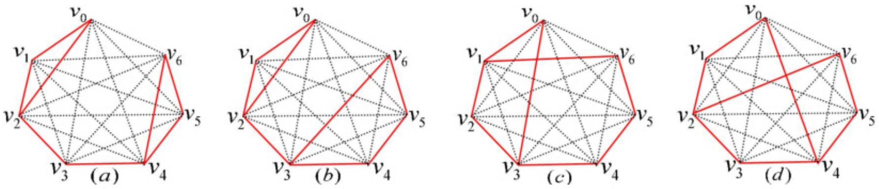

Figure 4: The graphs have five vertices with r-$deg(v_i) = 2$, b-$deg(v_i) = 4$, which contain a Hamiltonian path.

Lemma 5. *The set $\mathcal{S}$ corresponding to orthogonality graph which have five vertices with r-$deg(v_i) = 2$, b-$deg(v_i) = 4$ and the other two vertices with r-$deg(v_i) = 3$, b-$deg(v_i) = 3$ are always extendible.*

Proof. All the orthogonality graphs which have five vertices with r-$deg(v_i) = 2$, b-$deg(v_i) = 4$ and the other vertices with r-$deg(v_i) = 3$, b-$deg(v_i) = 3$, can be divided into three classes of graph: graphs containing a Hamiltonian cycle, graphs containing a Hamiltonian path, and disconnected graphs (there does not exist a connected graph containing no Hamiltonian path by lemma 4).

Case 1 (graphs containing a Hamiltonian cycle): In the sense of the same orthogonality graph, it contains two subcases Fig. 3(a)(b).

Case 1.1 (Fig. 3(a)): The method of proof is same as the case 1 (Fig. 2(a)) in the proof of lemma 3.

Case 1.2 (Fig. 3(b)): Considering the red square $v_0v_1v_2v_3$, then either $|\alpha_1\rangle = |\alpha_3\rangle$ or $|\alpha_0\rangle = |\alpha_2\rangle$. Whatever it is, it always contradicts with Fig. 3(b).

Case 2 (disconnected graphs Fig. 3(c)): The proof is similar to the case 2 (Fig. 2(b)) in the proof of lemma 3.

Case 3 (graphs containing a Hamiltonian path): All the cases can be divided into four subcases in Fig. 4. In the other cases the graph either contains a Hamiltonian cycle or is isomorphic to one of the graphs in Fig. 4.

Case 3.1 (Fig. 4(a)): A discussion similar to that in Fig. 2(a) can be applied here. Considering the linear relationship of three states $|\beta_0\rangle, |\beta_1\rangle, |\beta_2\rangle$ on Bob's side, according to the symmetry of the graph the only difference is that when $|\beta_0\rangle = |\beta_1\rangle$, we have $|\beta_5\rangle = |\beta_6\rangle$. It means that $rank\{|\beta_0\rangle, |\beta_1\rangle, |\beta_4\rangle, |\beta_5\rangle, |\beta_6\rangle\} \leq 3$. While $rank\{|\alpha_2\rangle, |\alpha_3\rangle\} \leq 2$, thus the set $\mathcal{S}$ is extendible.

Case 3.2 (Fig. 4(b)): Considering the red square $v_3v_4v_5v_6$, if $|\alpha_3\rangle = |\alpha_5\rangle$, then $|\alpha_5\rangle \perp |\alpha_2\rangle$. It contradicts with Fig. 4(b). If $|\alpha_4\rangle = |\alpha_6\rangle$, $rank\{|\alpha_4\rangle, |\alpha_5\rangle, |\alpha_6\rangle\} \leq 2$. Since $|\beta_5\rangle \perp \{|\beta_0\rangle, |\beta_1\rangle, |\beta_2\rangle, |\beta_3\rangle\}$, $rank\{|\beta_0\rangle, |\beta_1\rangle, |\beta_2\rangle, |\beta_3\rangle\} \leq 3$. So the set $\mathcal{S}$ is extendible.

Case 3.3 (Fig. 4(c)): In the graph, v_0, v_2, v_4,and v_6 form a black simple complete graph. It means that on Bob's side the four states $|\beta_0\rangle, |\beta_2\rangle, |\beta_4\rangle, |\beta_6\rangle$ are mutually orthogonal. Since $|\beta_5\rangle \perp \{|\beta_0\rangle, |\beta_2\rangle\}$, $|\beta_5\rangle$ can be linearly expressed by $|\beta_4\rangle$ and $|\beta_6\rangle$. (i) If $|\beta_6\rangle$ can be linearly expressed by $|\beta_4\rangle$ and $|\beta_5\rangle\}$, then $|\beta_1\rangle \perp |\beta_6\rangle$ since $|\beta_1\rangle \perp \{|\beta_4\rangle, |\beta_5\rangle\}$. It contradicts with Fig. 4(c). (ii) Otherwise, $|\beta_4\rangle = |\beta_5\rangle$. Since $|\beta_4\rangle \perp |\beta_6\rangle$, then $|\beta_5\rangle \perp |\beta_6\rangle$. It contradicts with Fig. 4(c).

Case 3.4 (Fig. 4(d)): Since on Bob's side $|\beta_0\rangle \perp \{|\beta_2\rangle, |\beta_3\rangle, |\beta_5\rangle, |\beta_6\rangle\}$, the four states are linearly dependent. If $|\beta_2\rangle$ can be linearly expressed by $\{|\beta_3\rangle, |\beta_5\rangle, |\beta_6\rangle\}$, then $|\beta_1\rangle \perp |\beta_2\rangle$ since $|\beta_1\rangle \perp \{|\beta_3\rangle, |\beta_5\rangle, |\beta_6\rangle\}$. It contradicts with Fig. 4(d). So $|\beta_2\rangle$ cannot be linearly expressed by $\{|\beta_3\rangle, |\beta_5\rangle, |\beta_6\rangle\}$, that is, $|\beta_3\rangle, |\beta_5\rangle$ and $|\beta_6\rangle$ are linearly dependent. Since $|\beta_3\rangle \perp \{|\beta_5\rangle, |\beta_6\rangle\}$, then $|\beta_5\rangle = |\beta_6\rangle$. While $|\beta_6\rangle \perp |\beta_4\rangle$, then $|\beta_5\rangle \perp |\beta_4\rangle$. It contradicts with Fig. 4(d).

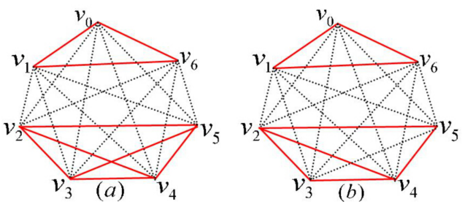

Figure 5:

According to above discussion, $\mathcal{S}$ satisfying the conditions of lemma are all extendible. That completes the proof. □

Lemma 6. *In orthogonality graph with seven vertices, if there only exist three vertices with r-deg*$(v_i) = 2$*, b-deg*$(v_i) = 4$ *and the other four vertices with r-deg*$(v_i) = 3$*, b-deg*$(v_i) = 3$*, the orthogonality graph must contain a red Hamiltonian path.*

Proof. We first show that the graph G is connected on Alice's side (only considering red edges). Similar to the proof of lemma 1. Suppose that it is disconnected, then there are only exists two connected components and the orthogonality graph is Fig. 5(a). However, as a orthogonality graph in $\mathbb{C}^3 \otimes \mathbb{C}^4$, it is impossible that there exist four states which are mutually orthogonal on Alice's side. So it is connected.

In the graph, there are four vertices with r-$deg(v_i) = 3$ and three vertices with r-$deg(v_i) = 2$. In order to use the lemma 4, we need to prove that (i) there are at least two vertices with r-$deg(v_i) = 3$ which are adjacent on Alice side; (ii) there exists an edge between two vertices r-$deg(v_i) = 3$ which is not a bridge.

Now we show that there are at least two vertices with r-$deg(v_i) = 3$ which are adjacent on Alice side. Suppose all the vertices with r-$deg(v_i) = 3$ are nonadjacent on Alice side, they are adjacent to vertices with r-$deg(v_i) = 2$. So a vertex with r-$deg(v_i) = 3$ must be adjacent to three vertices with r-$deg(v_i) = 2$. However, it is impossible because there are only three vertices with r-$deg(v_i) = 2$.

Next we present there exists an edge between two vertices r-$deg(v_i) = 3$ which is not a bridge. Suppose that the edges between two vertices r-$deg(v_i) = 3$ are all bridges. We delete one of the bridges e, then there are more than two connected components in $G - e$. Now there are two vertices with r-$deg(v_i) = 3$ and five vertices with r-$deg(v_i) = 2$. Then there are only exists two connected components in $G - e$ and the graph $G - e$ is Fig. 5(b). Obviously, there exists an edge between two vertices r-$deg(v_i) = 3$ which is not a bridge. It is a contradiction. So there exists an edge between two vertices r-$deg(v_i) = 3$ which are not a bridge. We delete the edge, then the graph $G - e$ still is connected. Hence the graph $G - e$ contains a Hamiltonian path by lemma 4. Thus the graph G must contain a red Hamiltonian path. □

Lemma 7. *The set $\mathcal{S}$ corresponding to orthogonality graph which have three vertices with r-deg*$(v_i) = 2$*, b-deg*$(v_i) = 4$ *and the other four vertices with r-deg*$(v_i) = 3$*, b-deg*$(v_i) = 3$ *are always extendible except Fig.3 in main body of the paper.*

Proof. Employing lemma 6 all the orthogonality graphs can be divided into two classes of graphs: graphs containing a red Hamiltonian cycle and graphs containing a red Hamiltonian path.

Case 1 (graphs containing a red Hamiltonian cycle): Since there are four vertices with r-$deg(v_i) = 3$, b-$deg(v_i) = 3$ in orthogonality graph containing a red Hamiltonian cycle, the case can be divided into five subcases:

Case 1.1 (Fig. 6(a)) and *Case 1.2* (Fig. 6(b)): The proof is similar to the case 2 (Fig. 1(b)) of lemma 2 and the case 1 (Fig. 2(a)) of lemma 3, respectively.

Case 1.3 (Fig. 6(c)), *Case 1.4* (Fig. 6(d)) and *Case 1.5* (Fig. 6(e)): Considering red squares, obviously, it contradicts with Fig. 6(c), Fig. 6(d) and Fig. 6(e), respectively.

Case 2 (graphs containing a red Hamiltonian path): In the sense of the same orthogonality graph, it can be divided into eight subcases as shown in Fig. 7. In other cases the graph either contains a Hamiltonian cycle or is isomorphic to one of the graphs in Fig. 7.

Case 2.1 (Fig. 7(a)), *Case 2.2* (Fig. 7(b)),*Case 2.5* (Fig. 7(e)), *Case 2.6* (Fig. 7(f)): Considering red squares, obviously, it contradicts with their graphs.

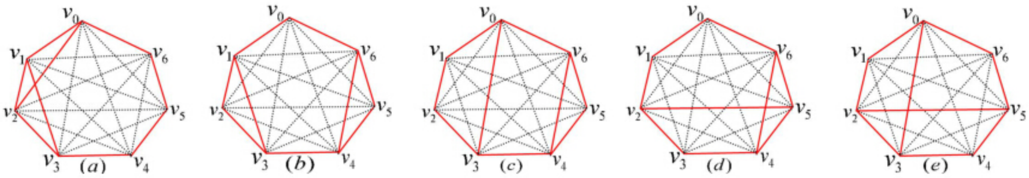

Figure 6: The graphs have three vertices with r-$deg(v_i) = 2$, b-$deg(v_i) = 4$, which contain a Hamiltonian cycle.

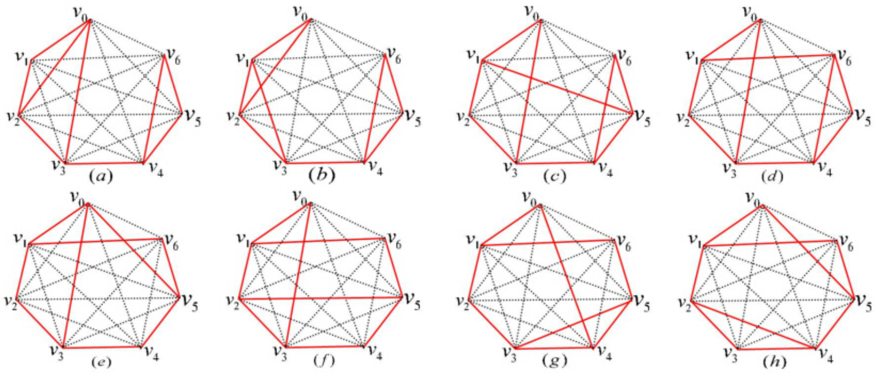

Figure 7: The graphs have three vertices with r-$deg(v_i) = 2$, b-$deg(v_i) = 4$, which contain a Hamiltonian path.

Case 2.3 (Fig. 7(c)) and *Case 2.4* (Fig. 7(d)): Considering the red square $v_0v_1v_2v_3$, if $|\alpha_1\rangle = |\alpha_3\rangle$, obviously, it contradicts with their graphs. So $|\alpha_0\rangle = |\alpha_2\rangle$. Then $rank\{|\alpha_0\rangle, |\alpha_1\rangle, |\alpha_2\rangle\} \leq 3$. Since $|\beta_0\rangle \perp |\beta_2\rangle$ and $\{|\beta_0\rangle, |\beta_2\rangle\} \perp \{|\beta_4\rangle, |\beta_5\rangle, |\beta_6\rangle\}$, then $rank\{|\beta_4\rangle, |\beta_5\rangle, |\beta_6\rangle\} \leq 2$. Thus $rank\{|\beta_3\rangle, |\beta_4\rangle, |\beta_5\rangle, |\beta_6\rangle\} \leq 3$. Furthermore, $\mathcal{S}$ is extendible.

Case 2.7 (Fig. 7(g)): The graph is Fig.3 in main body of the paper, which we try to find.

Case 2.8 (Fig. 7(h)): The similar discussion in Case 2.3 (Fig. 7(c)) for the red square $v_0v_1v_6v_5$ can be applied here.

Through the above discussion, $\mathcal{S}$ satisfying the conditions of lemma 7 is extendible except Fig.3 in main body of the paper. □

Proof of Theorem. It should be noted that all the figures we refer to in the following proof are the ones in main body of the paper.

Proof of Theorem 8.

Proof. We will present the distinguishability of UPBs by separable measurements, respectively.

Case 1 (Fig.2(a)): We first prove a property for six-state UPBs, *i.e.*, when any single state is removed from the six-state UPBs, the remaining states can be distinguished by LOCC. Considering the orthogonality graph Fig.2(a), without loss of generality, we assume that $|\psi_0\rangle$ is removed. Bob can distinguish between $\{|\psi_i\rangle\}_{i=1}^3$ and $|\psi_5\rangle$ by local projection that splits state $|\psi_4\rangle$ into two projected states. However, the projected state $|\psi_4\rangle$ is still orthogonal to $\{|\psi_i\rangle\}_{i=1}^3$ and $|\psi_5\rangle$. Therefore, Alice and Bob are left with distinguishing four or two orthogonal product states which can always be distinguished by LOCC. So the property holds, which means that the remaining states is completable by removing any state from the six-state UPBs. Employing Theorem 2 in Ref. [2], six-state UPBs can be distinguished by separable measurements.

Case 2 (Fig.4(b) and Fig.5(b)(c)(d)): It is straightforward to see that the four different UPBs are all from the extension of UPBs in $\mathbb{C}^3 \otimes \mathbb{C}^3$. We can use a same method to distinguish them by separable measurements. First, Bob can distinguish between $\{|\psi_i\rangle\}_{i=0}^2$ and $\{|\psi_i\rangle\}_{i=3}^7$ by local projection. And the orthogonality among post-measurement states $\{|\psi_i\rangle\}_{i=0}^2$ remains unchanged, so does the orthogonality among post-measurement

states $\{|\psi_i\rangle\}_{i=3}^7$. Since Alice and Bob can always distinguish the three or five orthogonal product states by separable measurements, the four different UPBs can be distinguished by separable measurements. $\square$

Proof of Theorem 9.

Proof. We first construct a UPB. Employing Eq.4 in main body of the paper, we first show the following UPB $\mathcal{S}_1 = \{|\psi_i\rangle = |\alpha_i^{(1)}\rangle \otimes |\beta_i^{(1)}\rangle\}_{i=0}^7$.

$$\begin{aligned}
|\psi_0\rangle &= |0\rangle(|0\rangle + \sqrt{2}|1\rangle); & |\psi_1\rangle &= |1\rangle|0\rangle;\\
|\psi_2\rangle &= |2\rangle[(2\cos x + \sqrt{3}\sin x)|0\rangle & |\psi_3\rangle &= (|0\rangle + |1\rangle)(|2\rangle + |3\rangle);\\
&+(2\sqrt{2}\cos x + \sqrt{3}\sin x)|1\rangle + \sqrt{6}\sin x|3\rangle]; & |\psi_4\rangle &= (|0\rangle - |1\rangle - |2\rangle)|2\rangle;\\
|\psi_5\rangle &= (|0\rangle + |2\rangle)[2|0\rangle - \sqrt{2}|1\rangle & |\psi_6\rangle &= |1\rangle|1\rangle;\\
&+(\sqrt{2}-1)|2\rangle - (\sqrt{2}-1)|3\rangle]; & |\psi_7\rangle &= |2\rangle(|0\rangle + |1\rangle + \sqrt{2}|3\rangle);
\end{aligned} \tag{1}$$

where $\sin x = -\sqrt{(37+24\sqrt{2})/217}$. The set of rank-one product projections [3]

$$\mathcal{P}_i = \{|\alpha\rangle\langle\alpha| \otimes |\beta\rangle\langle\beta| : |\alpha\rangle \in \mathbb{C}^3, |\beta\rangle \in \mathbb{C}^4, \langle\alpha|\alpha\rangle = \langle\beta|\beta\rangle = 1, \text{and} \quad |\alpha\rangle|\beta\rangle \perp \{|\psi_j\rangle\}_{j=0}^7 \backslash \{|\psi_i\rangle\}\}$$

for each $i = 0, \dots, 7$. Each element of $\mathcal{P}_i$ corresponds to a product state $|\alpha\rangle|\beta\rangle$, yielding a orthonormal product set. Here, we represent the element of $\mathcal{P}_i$ by product states $|\psi_{i,j}\rangle = |\alpha_{i,j}\rangle|\beta_{i,j}\rangle$. To be explicit, all the product states corresponding to $\{\mathcal{P}_i\}_{i=0}^7$ are as follows (normalization factors are omitted for brevity):

$$\begin{aligned}
|\psi_{0,1}\rangle &= |0\rangle(|0\rangle + \sqrt{2}|1\rangle); & |\psi_{1,1}\rangle &= |1\rangle|0\rangle;\\
|\psi_{2,1}\rangle &= |\psi_2\rangle = |2\rangle[(4\sqrt{2}-5)|0\rangle & |\psi_{3,1}\rangle &= |1\rangle|3\rangle;\\
&+(7-4\sqrt{2})|1\rangle - \sqrt{2}|3\rangle]; & |\psi_{3,2}\rangle &= (|0\rangle + 2|1\rangle - |2\rangle)|2\rangle;\\
|\psi_{3,3}\rangle &= (|0\rangle + |1\rangle)(|2\rangle + |3\rangle); & |\psi_{3,4}\rangle &= (|0\rangle - |2\rangle)[2|0\rangle - \sqrt{2}|1\rangle - (\sqrt{2}-1)|3\rangle];\\
|\psi_{3,5}\rangle &= (|0\rangle + |2\rangle)[2|0\rangle - \sqrt{2}|1\rangle & |\psi_{3,6}\rangle &= |0\rangle[2|0\rangle - \sqrt{2}|1\rangle + 6(\sqrt{2}+1)|2\rangle];\\
&-(5+7\sqrt{2})|2\rangle - (\sqrt{2}-1)|3\rangle]; & |\psi_{4,1}\rangle &= |1\rangle(|2\rangle - |3\rangle);\\
|\psi_{4,2}\rangle &= (|0\rangle - |1\rangle - |2\rangle)|2\rangle; & |\psi_{4,3}\rangle &= (|0\rangle - |1\rangle)(|2\rangle + |3\rangle);\\
|\psi_{4,4}\rangle &= (|0\rangle - |2\rangle)[2|0\rangle - \sqrt{2}|1\rangle & |\psi_{4,5}\rangle &= |2\rangle[2|0\rangle - \sqrt{2}|1\rangle - (5+7\sqrt{2})|2\rangle - (\sqrt{2}-1)|3\rangle];\\
&+(\sqrt{2}-1)|2\rangle - (\sqrt{2}-1)|3\rangle]; & |\psi_{4,6}\rangle &= |0\rangle[(2-2\sqrt{2})|0\rangle + (2-\sqrt{2})|1\rangle + 3|2\rangle - 3|3\rangle];\\
|\psi_{5,1}\rangle &= (|0\rangle - |1\rangle)|3\rangle; & |\psi_{5,2}\rangle &= (|0\rangle + |1\rangle)(|2\rangle - |3\rangle);\\
|\psi_{5,3}\rangle &= (|0\rangle - |1\rangle + 2|2\rangle)|2\rangle; & |\psi_{5,4}\rangle &= |2\rangle[2|0\rangle - \sqrt{2}|1\rangle - (\sqrt{2}-1)|3\rangle];\\
|\psi_{5,5}\rangle &= (|0\rangle + |2\rangle)[2|0\rangle - \sqrt{2}|1\rangle & |\psi_{5,6}\rangle &= |0\rangle(\sqrt{2}|0\rangle - |1\rangle);\\
&+(\sqrt{2}-1)|2\rangle - (\sqrt{2}-1)|3\rangle]; & |\psi_{6,1}\rangle &= |1\rangle|1\rangle;\\
|\psi_{7,1}\rangle &= |2\rangle(|0\rangle + |1\rangle + \sqrt{2}|3\rangle).
\end{aligned} \tag{2}$$

We can verify by a computer that the identity operator $\mathbb{1} \otimes \mathbb{1}$ is not in the linear span of the set. Thus the UPBs cannot be distinguished by separable measurements according to Theorem 7 in Ref. [3]. $\square$

Next we present that there are distinguishable UPBs by separable measurements in Fig.3, Fig.4(c) and Fig.5(a), respectively.

For Fig.3, we consider the UPB Eq.2 in main body of the paper. The set $\{|S_0\rangle, |S_1\rangle, |S_2\rangle, |L_0\rangle, |L_1\rangle, |L_2\rangle, |F\rangle\}$ is denoted as $\{|\psi_i\rangle\}_{i=0}^6$. All the product states corresponding to $\{\mathcal{P}_i\}_{i=0}^6$ are as follows:

$$
\begin{aligned}
&|\psi_{0,1}\rangle = |1\rangle(2|0\rangle - |2\rangle - |3\rangle); && |\psi_{1,1}\rangle = |2\rangle(|0\rangle - 2|1\rangle + |3\rangle);\\
&|\psi_{0,2}\rangle = |0\rangle(2|0\rangle - |1\rangle - |3\rangle); && |\psi_{1,2}\rangle = |1\rangle(2|1\rangle - |2\rangle - |3\rangle);\\
&|\psi_{0,3}\rangle = (|0\rangle - |1\rangle)|0\rangle; && |\psi_{1,3}\rangle = (|1\rangle - |2\rangle)|1\rangle;\\
&|\psi_{2,1}\rangle = |2\rangle(|0\rangle - 2|2\rangle + |3\rangle); && |\psi_{3,1}\rangle = |0\rangle(|1\rangle - |3\rangle);\\
&|\psi_{2,2}\rangle = |0\rangle(|1\rangle - 2|2\rangle + |3\rangle); && |\psi_{3,2}\rangle = (2|0\rangle - |1\rangle - |2\rangle)|1\rangle;\\
&|\psi_{2,3}\rangle = (|0\rangle - |2\rangle)|2\rangle; && |\psi_{3,3}\rangle = (|0\rangle + |1\rangle + |2\rangle)(|0\rangle - 3|1\rangle + |2\rangle + |3\rangle);\\
&|\psi_{4,1}\rangle = |1\rangle(|2\rangle - |3\rangle); && |\psi_{5,1}\rangle = |2\rangle(|0\rangle - |3\rangle);\\
&|\psi_{4,2}\rangle = (|0\rangle - 2|1\rangle + |2\rangle)|2\rangle; && |\psi_{5,2}\rangle = (|0\rangle + |1\rangle - 2|2\rangle)|0\rangle;\\
&|\psi_{4,3}\rangle = (|0\rangle + |1\rangle + |2\rangle)(|0\rangle + |1\rangle - 3|2\rangle + |3\rangle); && |\psi_{5,3}\rangle = (|0\rangle + |1\rangle + |2\rangle)(3|0\rangle - |1\rangle - |2\rangle - |3\rangle);\\
&|\psi_{6,1}\rangle = |2\rangle(|0\rangle + |3\rangle); && |\psi_{6,2}\rangle = |1\rangle(|2\rangle + |3\rangle);\\
&|\psi_{6,3}\rangle = (|1\rangle + |2\rangle)|1\rangle; && |\psi_{6,4}\rangle = |0\rangle(|1\rangle + |3\rangle;\\
&|\psi_{6,5}\rangle = (|0\rangle + |2\rangle)|2\rangle; && |\psi_{6,6}\rangle = (|0\rangle + |1\rangle)|0\rangle;\\
&|\psi_{6,7}\rangle = (|0\rangle + |1\rangle + |2\rangle)(|0\rangle + |1\rangle + |2\rangle + |3\rangle);
\end{aligned} \tag{3}
$$

The identity operator $\mathbb{1} \otimes \mathbb{1}$ can be written as a nonnegative linear combination of $\{|\psi_{i,j}\rangle\langle\psi_{i,j}|\}$, and nonzero coefficients $\lambda_{0,3} = \lambda_{1,3} = \lambda_{2,3} = \lambda_{3,1} = \lambda_{4,1} = \lambda_{5,1} = \lambda_{6,1} = \lambda_{6,2} = \lambda_{6,3} = \lambda_{6,4} = \lambda_{6,5} = \lambda_{6,6} = 1$, where $\lambda_{i,j}$ is the coefficient of $|\psi_{i,j}\rangle\langle\psi_{i,j}|$. So the UPB from Fig.3 can be distinguished by separable measurements.

It is worth noting that although normalization factors of $|\psi_{i,j}\rangle$ are omitted, $|\psi_{i,j}\rangle$ must be normalized when calculating the coefficients $\lambda_{i,j}$. In what follows, $|\psi_{i,j}\rangle$ should also be normalized, we will not repeat it again.

For Fig.4(c), we consider the following UPB $\mathcal{S}_3 = \{|\psi_i\rangle = |\alpha_i^{(3)}\rangle \otimes |\beta_i^{(3)}\rangle\}_{i=0}^7$.

$$
\begin{aligned}
&|\psi_0\rangle = |0\rangle|1\rangle; \quad |\psi_1\rangle = |1\rangle(|0\rangle + |1\rangle); \quad |\psi_2\rangle = |2\rangle(|0\rangle + |1\rangle); \quad |\psi_3\rangle = |0\rangle|0\rangle;\\
&|\psi_4\rangle = (|1\rangle + |2\rangle)(|0\rangle - |1\rangle + \sqrt{2}|3\rangle); \quad |\psi_5\rangle = (\sqrt{2}|0\rangle + |1\rangle - |2\rangle)(|2\rangle + |3\rangle);\\
&|\psi_6\rangle = [(1+\sqrt{2})|0\rangle - 2|1\rangle + \sqrt{2}|2\rangle]|2\rangle; \quad |\psi_7\rangle = (|1\rangle + \sqrt{2}|2\rangle)(|0\rangle - |1\rangle + \sqrt{2}|2\rangle - \sqrt{2}|3\rangle);
\end{aligned} \tag{4}
$$

All the product states corresponding to $\{\mathcal{P}_i\}_{i=0}^7$ are as follows:

$$
\begin{aligned}
&|\psi_{0,1}\rangle = |0\rangle|1\rangle; \quad |\psi_{1,1}\rangle = |1\rangle(|0\rangle + |1\rangle); && |\psi_{2,1}\rangle = |2\rangle(|0\rangle + |1\rangle); \quad |\psi_{3,1}\rangle = |0\rangle|0\rangle;\\
&|\psi_{4,1}\rangle = (|1\rangle + \sqrt{2}|2\rangle)(\sqrt{2}|0\rangle - \sqrt{2}|1\rangle - |2\rangle + |3\rangle); && |\psi_{4,2}\rangle = [(3\sqrt{2}-6)|0\rangle - \sqrt{2}|1\rangle + |2\rangle](|2\rangle - |3\rangle);\\
&|\psi_{4,3}\rangle = [(7\sqrt{2}-8)|0\rangle + (8\sqrt{2}+3)|1\rangle + 17|2\rangle](|2\rangle + |3\rangle); && |\psi_{4,4}\rangle = (|1\rangle + |2\rangle)(|0\rangle - |1\rangle + \sqrt{2}|3\rangle);\\
&|\psi_{4,5}\rangle = [(1+\sqrt{2})|0\rangle - 2|1\rangle + \sqrt{2}|2\rangle]|3\rangle; && |\psi_{4,6}\rangle = (\sqrt{2}|1\rangle - |2\rangle)(|0\rangle - |1\rangle);\\
&|\psi_{5,1}\rangle = |0\rangle|3\rangle; && |\psi_{5,2}\rangle = (\sqrt{2}|0\rangle + |1\rangle - |2\rangle)(|2\rangle + |3\rangle);\\
&|\psi_{5,3}\rangle = (|1\rangle + \sqrt{2}|2\rangle)(|0\rangle - |1\rangle - 2\sqrt{2}|2\rangle - \sqrt{2}|3\rangle); && |\psi_{5,4}\rangle = [(6-3\sqrt{2})|0\rangle + \sqrt{2}|1\rangle - |2\rangle]|2\rangle;\\
&|\psi_{5,5}\rangle = (|1\rangle - |2\rangle)(|0\rangle - |1\rangle + \sqrt{2}|3\rangle); && |\psi_{5,6}\rangle = (\sqrt{2}|1\rangle - |2\rangle)(|0\rangle - |1\rangle - \sqrt{2}|3\rangle);\\
&|\psi_{6,1}\rangle = |0\rangle(|2\rangle - |3\rangle); && |\psi_{6,2}\rangle = (\sqrt{2}|0\rangle - |1\rangle + |2\rangle)(|2\rangle + |3\rangle);\\
&|\psi_{6,3}\rangle = (|1\rangle - |2\rangle)(\sqrt{2}|0\rangle - \sqrt{2}|1\rangle - |2\rangle + |3\rangle); && |\psi_{6,4}\rangle = (|1\rangle + |2\rangle)(|0\rangle - |1\rangle - 2\sqrt{2}|2\rangle - \sqrt{2}|3\rangle);\\
&|\psi_{6,5}\rangle = (\sqrt{2}|1\rangle - |2\rangle)(|0\rangle - |1\rangle + \sqrt{2}|2\rangle - \sqrt{2}|3\rangle); && |\psi_{6,6}\rangle = [(1+\sqrt{2})|0\rangle - 2|1\rangle + \sqrt{2}|2\rangle]|2\rangle;\\
&|\psi_{7,1}\rangle = (|1\rangle - |2\rangle)(|0\rangle - |1\rangle); && |\psi_{7,2}\rangle = (|1\rangle + |2\rangle)(|0\rangle - |1\rangle - \sqrt{2}|3\rangle);\\
&|\psi_{7,3}\rangle = (|1\rangle + \sqrt{2}|2\rangle)(|0\rangle - |1\rangle + \sqrt{2}|2\rangle - \sqrt{2}|3\rangle); && |\psi_{7,4}\rangle = (\sqrt{2}|0\rangle + |1\rangle - |2\rangle)(|2\rangle - |3\rangle);\\
&|\psi_{7,5}\rangle = [\sqrt{2}|0\rangle + (4\sqrt{2}+5)|1\rangle + (4\sqrt{2}+7)|2\rangle]|2\rangle; && |\psi_{7,6}\rangle = (\sqrt{2}|0\rangle - |1\rangle + |2\rangle)|3\rangle;
\end{aligned} \tag{5}
$$

We can verify that the identity operator $\mathbb{1} \otimes \mathbb{1}$ can be written as a nonnegative linear combination of $\{|\psi_{i,j}\rangle\langle\psi_{i,j}|\}$, where nonzero coefficients $\lambda_{0,1} = \lambda_{1,1} = \lambda_{2,1} = \lambda_{3,1} = \lambda_{4,4} = \lambda_{5,2} = \lambda_{6,1} = \lambda_{6,2} = \lambda_{6,3} = \lambda_{6,4} = \lambda_{6,5} = \lambda_{7,3} = 1$. Thus the UPB from Fig.4(c) can be distinguished by separable measurements.

For Fig.5(a), we consider the UPB $\mathcal{S}_{13} = \{|\psi_i\rangle = |\alpha_i^{(1)}\rangle \otimes |\beta_{i-2mod8}^{(3)}\rangle\}_{i=0}^7$, where $|\alpha_i^{(1)}\rangle \in \mathcal{S}_1$ and $|\beta_i^{(3)}\rangle \in$

$\mathcal{S}_3$. The states $|\psi_i\rangle$ are as follows:

$$\begin{array}{ll} |\psi_0\rangle = |0\rangle(|0\rangle + |1\rangle); & |\psi_1\rangle = |1\rangle|0\rangle; \\ |\psi_2\rangle = |2\rangle(|0\rangle - |1\rangle + \sqrt{2}|3\rangle); & |\psi_3\rangle = (|0\rangle + |1\rangle)(|2\rangle + |3\rangle); \\ |\psi_4\rangle = (|0\rangle - |1\rangle - |2\rangle)|2\rangle; & |\psi_5\rangle = (|0\rangle + |2\rangle)(|0\rangle - |1\rangle + \sqrt{2}|2\rangle - \sqrt{2}|3\rangle); \\ |\psi_6\rangle = |1\rangle|1\rangle; & |\psi_7\rangle = |2\rangle(|0\rangle + |1\rangle); \end{array} \tag{6}$$

All the product states corresponding to $\{\mathcal{P}_i\}_{i=0}^{7}$ are as follows:

$$\begin{array}{ll} |\psi_{0,1}\rangle = |0\rangle(|0\rangle + |1\rangle); & |\psi_{1,1}\rangle = |1\rangle|0\rangle; \\ |\psi_{2,1}\rangle = (|0\rangle - |2\rangle)(|0\rangle - |1\rangle); & |\psi_{2,2}\rangle = |2\rangle(|0\rangle - |1\rangle + \sqrt{2}|3\rangle); \\ |\psi_{2,3}\rangle = (|0\rangle - |1\rangle - |2\rangle)|3\rangle; & |\psi_{2,4}\rangle = (|0\rangle + 2|1\rangle - |2\rangle)(|2\rangle - |3\rangle); \\ |\psi_{2,5}\rangle = (|0\rangle - |1\rangle + 2|2\rangle)(|2\rangle + |3\rangle); & |\psi_{2,6}\rangle = (|0\rangle + |2\rangle)(\sqrt{2}|0\rangle - \sqrt{2}|1\rangle - |2\rangle + |3\rangle); \\ |\psi_{3,1}\rangle = |1\rangle|3\rangle; & |\psi_{3,2}\rangle = (|0\rangle + |1\rangle)(|2\rangle + |3\rangle); \\ |\psi_{3,3}\rangle = (|0\rangle + 2|1\rangle - |2\rangle)|2\rangle; & |\psi_{3,4}\rangle = (|0\rangle - |2\rangle)(|0\rangle - |1\rangle - \sqrt{2}|3\rangle); \\ |\psi_{3,5}\rangle = (|0\rangle + |2\rangle)(|0\rangle - |1\rangle - 2\sqrt{2}|2\rangle - \sqrt{2}|3\rangle); & |\psi_{3,6}\rangle = |0\rangle(|0\rangle - |1\rangle + \sqrt{2}|3\rangle); \\ |\psi_{4,1}\rangle = |1\rangle(|2\rangle - |3\rangle); & |\psi_{4,2}\rangle = (|0\rangle - |1\rangle)(|2\rangle + |3\rangle); \\ |\psi_{4,3}\rangle = (|0\rangle - |1\rangle - |2\rangle)|2\rangle; & |\psi_{4,4}\rangle = |2\rangle(|0\rangle - |1\rangle - 2\sqrt{2}|2\rangle - \sqrt{2}|3\rangle); \\ |\psi_{4,5}\rangle = |0\rangle(\sqrt{2}|0\rangle - \sqrt{2}|1\rangle - |2\rangle + |3\rangle); & |\psi_{4,6}\rangle = (|0\rangle - |2\rangle)(|0\rangle - |1\rangle + \sqrt{2}|2\rangle - \sqrt{2}|3\rangle); \\ |\psi_{5,1}\rangle = |0\rangle(|0\rangle - |1\rangle); & |\psi_{5,2}\rangle = |2\rangle(|0\rangle - |1\rangle - \sqrt{2}|3\rangle); \\ |\psi_{5,3}\rangle = (|0\rangle + |2\rangle)(|0\rangle - |1\rangle + \sqrt{2}|2\rangle - \sqrt{2}|3\rangle); & |\psi_{5,4}\rangle = (|0\rangle - |1\rangle)|3\rangle; \\ |\psi_{5,5}\rangle = (|0\rangle + |1\rangle)(|2\rangle - |3\rangle); & |\psi_{5,6}\rangle = (|0\rangle - |1\rangle + 2|2\rangle)|2\rangle; \\ |\psi_{6,1}\rangle = |1\rangle|1\rangle; & |\psi_{7,1}\rangle = |2\rangle(|0\rangle + |1\rangle); \end{array} \tag{7}$$

The identity operator $\mathbb{1} \otimes \mathbb{1}$ can be written as a nonnegative linear combination of $\{|\psi_{i,j}\rangle\langle\psi_{i,j}|\}$, where nonzero coefficients $\lambda_{0,1} = \lambda_{1,1} = \lambda_{2,2} = \lambda_{3,2} = \lambda_{4,3} = \lambda_{5,1} = \lambda_{5,2} = \lambda_{5,4} = \lambda_{5,5} = \lambda_{5,6} = \lambda_{6,1} = \lambda_{7,1} = 1$. Thus the UPB from Fig.5(a) can be distinguished by separable measurements.

References

[1] Harris, J. M., Hirst, J. L. & Mossinghoff, M. J. *Combinatorics and Graph Theory* 2nd edn, (eds Axler, S. & Ribet, K.A.) Ch. 1, 1-72 (Springer, New York, 2008).

[2] DiVincenzo, D. P., Mor, T., Shor, P. W., Smolin, J. A. & Terhal, B. M. Unextendible product bases, uncompletable product bases and bound entanglement. *Commun. Math. Phys.* **238** 379-410 (2003).

[3] Bandyopadhyay, S. *et al.* Limitations on separable measurements by convex optimization. arXiv:1408.6981v1.
